# A Novel Artificial Neural Network Prognostic Model Based on a Cancer-Associated Fibroblast Activation Score System in Hepatocellular Carcinoma

**DOI:** 10.3389/fimmu.2022.927041

**Published:** 2022-07-08

**Authors:** Yiqiao Luo, Huaicheng Tan, Ting Yu, Jiangfang Tian, Huashan Shi

**Affiliations:** ^1^ Cancer Center, West China Hospital, Sichuan University, Chengdu, China; ^2^ West China School of Medicine, West China Hospital, Chengdu, China; ^3^ Department of Pathology, State Key Laboratory of Biotherapy, West China Hospital, West China School of Medicine, Sichuan University, Chengdu, China; ^4^ Department of Biotherapy, Cancer Center, West China Hospital, Sichuan University, Chengdu, China

**Keywords:** cancer-associated fibroblasts, hepatocellular carcinoma, artificial neural network, single-cell transcriptome analysis, prognosis

## Abstract

**Introduction:**

Hepatocellular carcinoma (HCC) ranks fourth as the most common cause of cancer-related death. It is vital to identify the mechanism of progression and predict the prognosis for patients with HCC. Previous studies have found that cancer-associated fibroblasts (CAFs) promote tumor proliferation and immune exclusion. However, the information about CAF-related genes is still elusive.

**Methods:**

The data were obtained from The Cancer Genome Atlas, International Cancer Genome Consortium, and Gene Expression Omnibus databases. On the basis of single-cell transcriptome and ligand–receptor interaction analysis, CAF-related genes were selected. By performing Cox regression and random forest, we filtered 12 CAF-related prognostic genes for the construction of the ANN model based on the CAF activation score (CAS). Then, functional, immune, mutational, and clinical analyses were performed.

**Results:**

We constructed a novel ANN prognostic model based on 12 CAF-related prognostic genes. Cancer-related pathways were enriched, and higher activated cell crosstalk was identified in high-CAS samples. High immune activity was observed in high-CAS samples. We detected three differentially mutated genes (*NBEA*, *RYR2*, and *FRAS1*) between high- and low-CAS samples. In clinical analyses, we constructed a nomogram to predict the prognosis of patients with HCC. 5-Fluorouracil had higher sensitivity in high-CAS samples than in low-CAS samples. Moreover, some small-molecule drugs and the immune response were predicted.

**Conclusion:**

We constructed a novel ANN model based on CAF-related genes. We revealed information about the ANN model through functional, mutational, immune, and clinical analyses.

## Introduction

According to epidemiologic data, hepatocellular carcinoma (HCC) accounts for more than 80% of primary liver cancers and is the fourth most common cause of cancer-related death worldwide (1, 2). To our knowledge, the treatment options are limited for patients with advanced HCC (3). Thus, it is important to understand the mechanism of the progression of HCC and predict the survival rate and novel small-molecule drugs for patients with HCC.

In the tumor microenvironment (TME), the crosstalk between tumorigenic cells and fibroblasts may be the cause of the emergence of hyperactive fibroblasts, which are called cancer-associated fibroblasts (CAFs) (4). CAFs have been verified to be tumor-promoting components that can secrete growth factors, inflammatory ligands, and extracellular matrix (ECM) proteins to promote tumor proliferation and immune exclusion (5).

The artificial neural network (ANN), which was introduced in the 1950s, is a machine learning technique inspired by the human neuronal synapse system (6). Previous studies have verified that the ANN model has a better predictive capacity than the logistic Cox regression model (7). Thus, the ANN model has been widely applied in the biochemical and medical fields (8, 9).

In our study, we performed not only single-cell transcriptome analysis of HCC but also ligand–receptor interactions to determine CAF-related genes. Through Cox regression and random forest analyses, we filtered 12 CAF-related prognostic genes, which were recruited to construct a prognostic ANN model. We further performed functional, immune, mutational, and clinical analyses to estimate the constructed ANN model thoroughly.

## Methods

### Data Preparation

The transcriptome RNA sequencing data, Illumina human methylation 450 cohort, copy number variation (CNV), and the corresponding related data of HCC were extracted from The Cancer Genome Atlas (TCGA) database (https://portal.gdc.cancer.gov/) (including 340 patients) and the International Cancer Genome Consortium (ICGC) data portal (https://dcc.icgc.org) (including 226 patients). The GSE76427 cohort was downloaded from the Gene Expression Omnibus (GEO) (https://www.ncbi.nlm.nih.gov/geo/) (including 115 patients). Patients with complete clinical information (stage, follow-up information, age, and gender) were selected for this study. Otherwise, the patients who did not meet the criteria were excluded.

### Single-Cell Transcriptome Analysis

The expression profiling of single-cell RNA sequence GSE151530 (10X Genomics), which contained 46 HCC samples, was obtained from the GEO database (10). We used the R package “Seurat” to analyze the single-cell RNA sequence data. We collected 47,822 cells for further analyses. The data were normalized by using “NormalizeData” and “ScaleData” from the “Seurat” R package. We divided the cells into six subclusters based on the annotation in GSE151530. The R package “monocle” was used to perform single-cell trajectory analysis (11). The cells were filtered with the following conditions: a) num_cells_expressed ≥ 10 and b) min_expr = 0.1. Subsequently, the top 1,500 variable genes were selected to perform a single-cell trajectory analysis. The R package “monocle” was used to visualize the trajectory.

### Cell Communication Analyses

The R package “Cellphonedb” was utilized to speculate the ligand–receptor pairs (P < 0.05) by Python. The crosstalk of ligand–receptor pairs between CAFs and other subclusters, as well as the activated pathways of cell communication, were analyzed by the R package “cellchat”.

### Functional Analyses

The gene annotation and analysis resource Metascape (https://metascape.org) was used for the enrichment analysis. After we obtained the differentially expressed genes, we performed gene ontology (GO) analyses by using Metascape. We used the gene enrichment analysis (GSEA) (4.1.0) application to obtain the enrichment pathways in high- and low–CAF activation score (CAS) samples. To analyze the functional enrichment of tumor-infiltrating immune cells (TICs), we calculated the relative abundance of immune cells in each sample by using the R package “cibersortR”. We obtained the immune-related pathways from a previous article (12). Angiogenesis, the T effector/IFN response, checkpoint, myeloid inflammation, epithelial-mesenchymal transition (EMT), and hypoxia were identified in previously published articles (13–15). The CTA, neoantigen, and proliferation scores were obtained in a previous article (16). Then, we performed ssGSEA to assess the enrichment score of samples by using the R package “gsva”.

### Mutational Analyses

We extracted the mutation data of HCC from the TCGA database by the R package “TCGAbiolinks”. The mutation data were further analyzed, and the mutational landscape and lollipop chart were illustrated by the R package “maftools”.

### Construction of a CAF-Related Prognostic ANN Model

We constructed and trained the ANN in the TCGA dataset by using the R package “survivalmodels” (https://cran.r-project.org/web/packages/survivalmodels/). The clinical data of HCC were extracted, and we performed univariate and multivariate Cox regression analyses by the R package “survival”. As a result, we obtained 14 candidates. Then, we performed a random forest (ntree = 1,000) to further filter our candidates. Finally, the R package “survivalmodel” was used to construct the ANN model. Twelve CAF-related prognostic genes were selected and input into the input layer. The activation function was ReLU in three hidden layers. The loss function was the negative log partial likelihood under the Cox PH model. The dropout parameter was used to avoid overfitting. We performed a 1,000 iteration random search using the adam optimizer utilizing “mlr3” packages to tune these hyperparameters. The CAS was calculated on the basis of Cox regression.

### Validation of the Constructed CAF-Related Prognostic ANN Model

The TCGA dataset was set as the training cohort, whereas ICGC and GSE76427 were used as the testing cohorts. The concordance index (C-index) was calculated using the R package “Pec”. The heatmap was illustrated by the R package “pheatmap”. The area under the curve (AUC) was calculated by using the R package “timeROC”. We performed Kaplan–Meier analyses in the three cohorts using the R package “survival”.

### Prediction of the Sensitivity of Chemotherapeutic Drugs and Exploration of Novel Small-Molecule Drugs

The Genomics of Drug Sensitivity in Cancer (GDSC) database (www.cancerRxgene.org), where we can obtain drug response data and genomic markers of sensitivity, was used to predict the sensitivity of four common chemotherapeutic drugs in the high- and low-CAS samples. We performed a ridge regression analysis to determine the half-maximal inhibitory concentration (IC50) by using the R package “pRRophetic”. To predict novel small-molecule drugs, we introduced two online databases: a) the Cancer Therapeutics Response Portal (CTRP) 2.0 database (http://portals.broadinstitute.org/ctrp/), which includes sensitivity data of 481 small-molecule compounds in 860 cancer cell lines (CCLs); and b) the Profiling Relative Inhibition Simultaneously in Mixtures (PRISM) database (https://www.theprismlab.org/), with which we can screen thousands of drugs in hundreds of human CCLs. The AUC is a standard value for the evaluation of drug sensitivity. A lower AUC value represents better drug sensitivity. In addition, the differentially expressed genes between high- and low-CAS samples of HCC were potential therapeutic targets. Thus, we detected potential drugs that targeted the genes and illustrated the corresponding mechanism of action (MoA) by using the online database ConnectivityMap (cMap) (https://clue.io/).

### Prediction of the Immunotherapeutic Response

We introduced the online database Tumor Immune Dysfunction and Exclusion (TIDE) (http://tide.dfci.harvard.edu) (17), which is a popular enrichment algorithm extensively used in cancer-related studies (18–20). We extracted the response to the treatment against PD-1 and CTLA4 in 47 patients (21) to predict the immunotherapeutic response between patients with HCC with high and low CAS based on subclass mapping (https://cloud.genepattern.org/gp/).

### Statistical Analyses

R software (version 4.0.4) was used to analyze statistical data and construct images. We used the Wilcoxon test to analyze the differences between the two groups. The difference in proportions was analyzed by the chi-squared test. A P-value < 0.05 was considered to be statistically significant. All correlation analyses were performed by Pearson’s correlation. The heatmap in our study was generated by the R package “pheatmap”. Univariate and multivariate Cox regression analyses were performed by the R package “survival”. The nomogram was built by using the R package “RMS”. The calibration curves and AUCs were obtained by the R packages “rms” and “survivalROC”. The 1-, 3-, and 5-year decision curve analysis (DCA) was performed by using the R package “rmda”. *P < 0.05, **P < 0.01, and ***P < 0.001.

## Results

### Single-Cell Transcriptome Analysis of HCC and the Functional Enrichment of CAF-Related Genes

**Figure 1:**
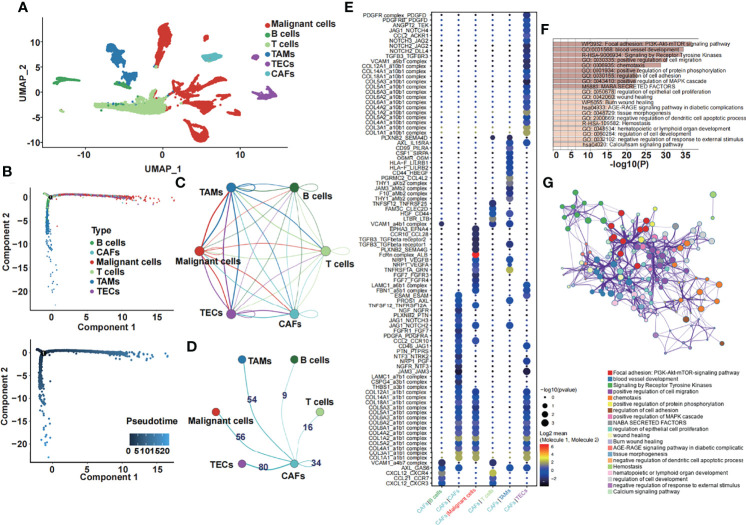
Single-cell transcriptome analysis and the function of CAF-related genes. **(A)** The six cell subclusters are shown. **(B)** Pseudotime analysis of six cell subclusters was performed. **(C)** The ligand–receptor interaction network among six subclusters is shown. **(D)** The number of ligand–receptor pairs between CAFs and other subclusters is shown. **(E)** The specific ligand–receptor pair between CAFs and other subclusters is shown. **(F)** Enrichment analysis of the differentially expressed CAF-related genes. **(G)** The corresponding network of the enrichment analysis of the differentially expressed CAF-related genes.

Six subclusters of single cells, including malignant cells, B and T cells, tumor-associated macrophages (TAMs), tumor-associated endothelial cells (TECs), and cancer-associated fibroblasts (CAFs), were split and illustrated by performing a uniform manifold approximation and projection (UMAP) plot (**Figure 1A**). Then, we used Monocle 2 to perform pseudotime analysis, which is a great approach to study lineage specification and hierarchize molecular events (22). We noticed that CAFs, which we were most interested in, were present at the end of the differentiation trajectory (**Figure 1B**). Furthermore, we performed the ligand–receptor interaction network among six subclusters (**Figure 1C**) and extracted the number of ligand–receptor pairs between CAFs and other subclusters (**Figure 1D**). We demonstrated that CAFs and TECs had the most ligand–receptor pairs, followed by CAFs and TAMs. Subsequently, we performed ligand–receptor interactions between CAFs and other subclusters (**Figure 1E**). Genes that were significantly related to CAFs were chosen for further analysis. Functional enrichment analysis of the CAF-related genes was performed by the online enrichment analysis tool Matascape in a bar graph (**Figure 1F**) and corresponding network (**Figure 1G**). Tumor-associated pathways were enriched, such as the PI3K-Akt-mTOR signaling pathway, blood vessel development, signaling by receptor tyrosine kinases, and positive regulation of cell migration.

### Twelve CAF-Related Genes Were Identified as a Predictive Model in HCC

**Figure 2:**
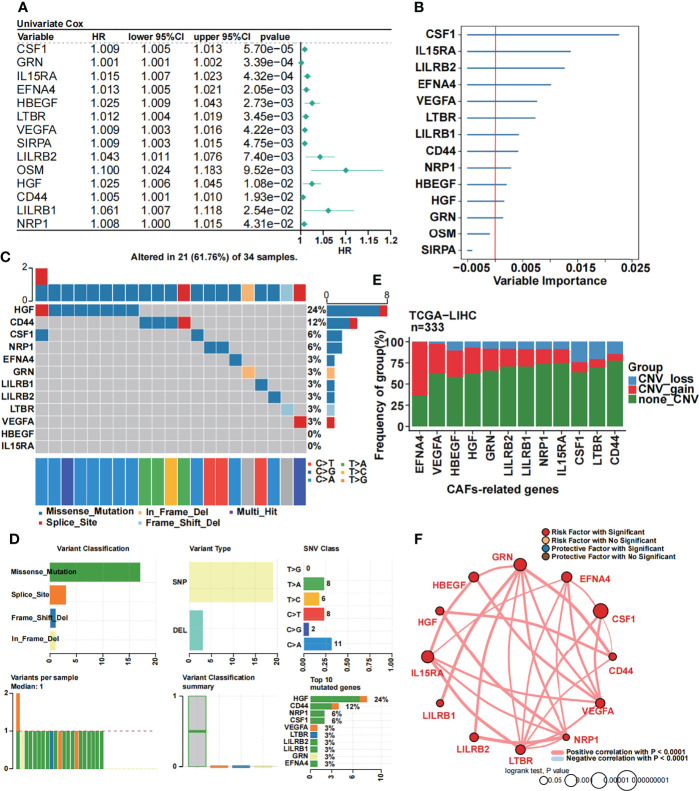
Identification of 12 CAF-related prognostic genes. **(A)** Univariate Cox regression of CAF-related genes. **(B)** Random forest analysis of the candidate CAF-related prognostic genes. **(C)** The mutational landscape of 12 CAF-related prognostic genes. **(D)** Summary of the mutational analysis including variant classification, variant type, SNV class, variants per sample, variant classification summary, and the top 10 mutated genes. **(E)** The CNV status in 12 CAF-related prognostic genes. **(F)** Univariate Cox regression analysis and Pearson’s correlation of the 12 CAF-related prognostic genes.

We performed univariate Cox regression analysis to further screen CAF-related prognostic genes in the TCGA dataset. As a result, 14 genes were selected (**Figure 2A**). To further obtain the strictest model, we performed random forest analysis and filtered the candidate genes. Finally, 12 CAF-related genes with variable importance values greater than 0 were selected as a prognostic model for patients with HCC (**Figure 2B**). Then, we performed the mutational landscape of 12 CAF-related prognostic genes (**Figure 2C**). We demonstrated that *HGF* had the highest mutation (24%), followed by *CD44* (12%) and *CSF1* and *NRP1* (6%). The highest type of mutation was a missense mutation. Moreover, we summarized the mutation analysis results (**Figure 2D**). The most common variant and variant type were missense mutation and single-nucleotide polymorphisms (SNPs), respectively. The number of single-nucleotide variants (SNVs) showed that the cytosine (C) to adenine (A) mutation was the most frequent mutation. The median variant per sample was 1. Moreover, we listed the top 10 mutated genes for further analysis. Subsequently, we performed a CNV analysis of 12 CAF-related prognostic genes in the TCGA dataset (**Figure 2E**), and we found that *EFNA4* had the highest CNV gain mutation, whereas *CSF1* had the highest CNV loss mutation. Then, we constructed a circle plot to exhibit the correlation among 12 CAF-related prognostic genes (**Figure 2F**). The 12 CAF-related prognostic genes were all risk factors and had strong positive correlations with a P-value less than 0.0001.

### An ANN Prognostic Model Was Created on the Basis of 12 CAF-Related Prognostic Genes

**Figure 3:**
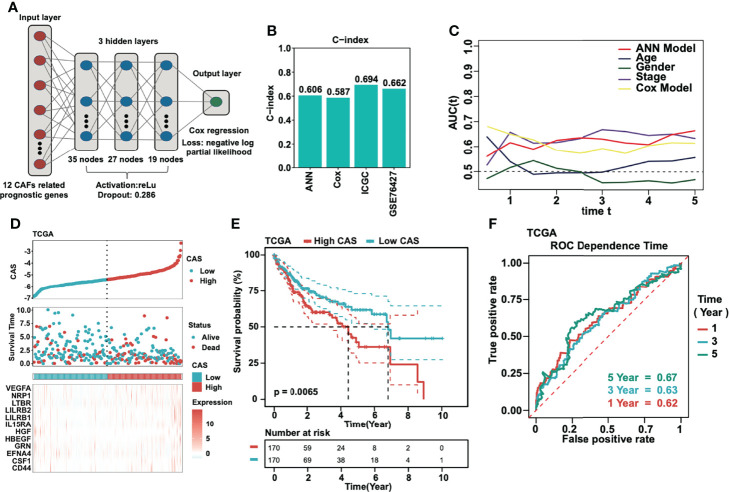
Construction of the ANN model. **(A)** The schematic diagram of the constructed ANN model. **(B)** Detection of the C-index of the ANN, traditional Cox model, ICGC, and GSE76427. **(C)** AUC analyses of the constructed ANN model compared to the traditional Cox model and other parameters. **(D)** The CAS and corresponding survival status in each sample in the TCGA dataset. **(E)** Kaplan–Meier analysis of high- and low-CAS samples in the TCGA dataset. **(F)** ROC analysis of the ANN model in 1-, 3-, and 5-year survival prediction in the TCGA dataset.

We constructed an ANN model based on the 12 selected CAF-related prognostic genes in the TCGA dataset. A schematic diagram is shown in **Figure 3A**. Twelve CAF-related genes were input into the input layer. The hyperparameters of the networks were as follows: a) three hidden layers; b) 35, 27, and 19 nodes in each layer; c) dropout rate = 0.286; d) learning rate = 0.4621984; and e) weight decay = 0.3156897. As a result, we obtained the output data. The output layer included one neuron and the CAS was calculated by performing Cox regression. To assess the prediction capacity, we introduced the C-index. We demonstrated that the C-index was higher in the ANN model than in the Cox model. Moreover, the C-index was satisfactory in the ICGC and GSE76427 datasets (**Figure 3B**). By performing AUC analysis, we obtained the same result that the ANN model was better than the Cox model (**Figure 3C**). Subsequently, the CAS of each sample was calculated in the TCGA dataset (**Figure 3D**), and patients with high CAS had a worse survival status, and vice versa. The result was confirmed in the ICGC and GSE76427 datasets (**Supplementary Figures 1A, B**). By performing Kaplan–Meier analysis in the TCGA dataset, we revealed that patients with HCC with high CAS had shorter overall survival (P = 0.0065) (**Figure 3E**). The result was verified in the ICGC and GSE76427 datasets (**Supplementary Figures 1C, D**), and the P-value was 0.016 in both datasets. To evaluate the accuracy of our ANN model, we performed ROC analysis in the TCGA, ICGC, and GSE76427 datasets. We illustrated that the 1-, 3-, and 5-year AUCs were more than 0.6 in the TCGA dataset (**Figure 3F**), which revealed that our ANN model was an accurate prognostic model. The result was also confirmed in the ICGC and GSE76427 datasets with an AUC less than 0.6 (**Supplementary Figures 1E, F**).

We performed univariate Cox regression subgroup analyses of the CAS in three datasets (TCGA, ICGC, and GSE76427). In the TCGA dataset, stage I, female, and age < 60 were considered the risk factors (**Supplementary Figure 2A**). In the ICGC dataset, stage III, stage IV, female, male, and age < 70 were regarded as the risk factors (**Supplementary Figure 2B**). In GSE76427, stage II and male were considered to be the risk factors (**Supplementary Figure 2C**).

### A Nomogram Was Constructed Based on our CAF-Related ANN Model

**Figure 4:**
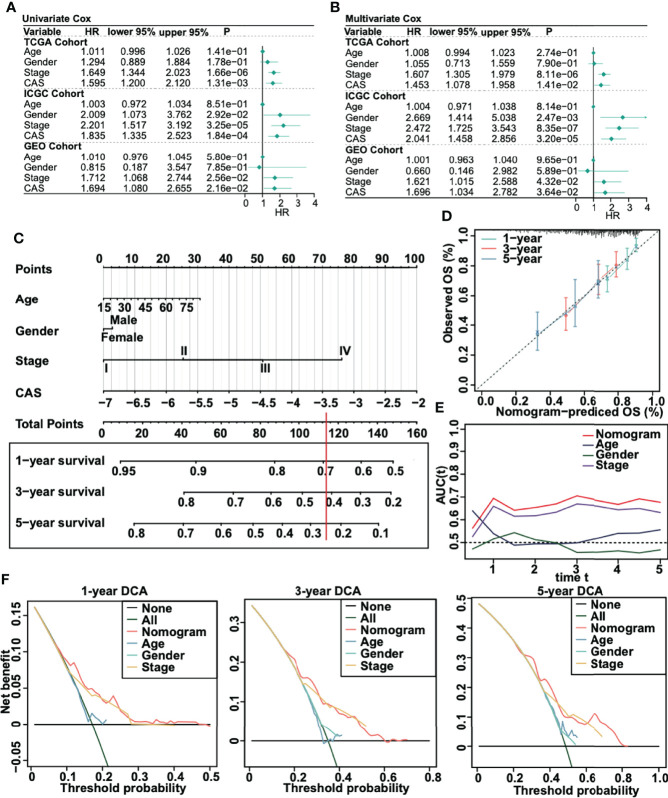
Creation of a nomogram for patients with HCC. **(A)** Univariate Cox regression in TCGA, ICGC, and GSE75427 cohorts. **(B)** Multivariate Cox regression in TCGA, ICGC, and GSE75427 cohorts. **(C)** Constructed nomogram based on CAS. **(D)** One-, 3-, and 5-year calibration curves were generated. **(E)** AUC analysis between the constructed nomogram and parameters was performed. **(F)** One-, 3-, and 5-year DCA was performed.

To evaluate whether our ANN model could act as an independent prognostic marker, we performed univariate and multivariate Cox regression analyses in three cohorts (TCGA, ICGC, and GSE76427) (**Figures 4A, B**). According to the univariate and multivariate Cox regression, the CAS of our ANN model was significantly associated with low overall survival in the three cohorts, which revealed that our constructed ANN model could act as an independent prognostic marker for patients with HCC. Thus, we built a nomogram based on the CAS to predict the 1-, 3-, and 5-year overall survival for patients with HCC (**Figure 4C**). For instance, a 60-year-old (20 points) male (3 points) patient with HCC with stage III (51 points) and −5 CAS values (40 points) received a total of 114 points, and the 1-, 3-, and 5-year survival rates of this patient were approximately 69%, 42%, and 25%, respectively. Then, we built calibration curves for assessing predicted risk versus observed risk (**Figure 4D**). The 1-, 3-, and 5-year calibration curves showed a great capacity for prediction. In addition, we calculated the AUC of our nomogram (**Figure 4E**), which indicated that the nomogram had the highest AUC compared to a single parameter. Finally, we performed 1-, 3-, and 5-year DCA to assess whether our constructed model was worth utilizing (**Figure 4F**). The results illustrated that our nomogram was acceptable for patients with HCC.

### Enrichment Analysis of the Constructed CAF-Related ANN Model

**Figure 5:**
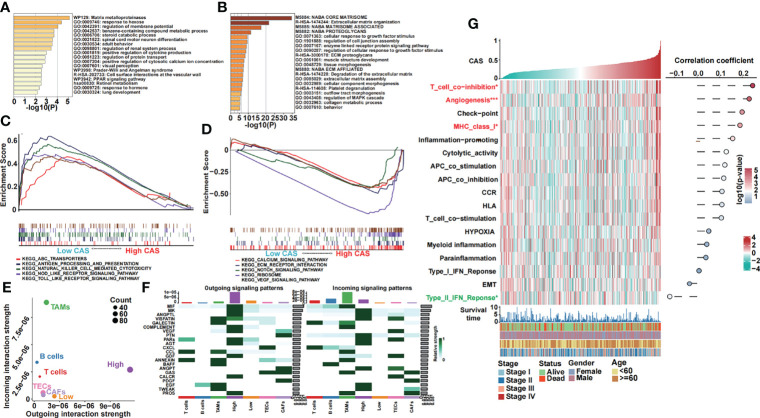
Functional analyses of our constructed ANN model. **(A)** GO analysis of differentially expressed genes in high-CAS samples was performed in Metascape. **(B)** GO analysis of differentially expressed genes in low-CAS samples was performed in Metascape. **(C)** Five KEGG pathways were enriched in the low-CAS samples by performing GSEA. **(D)** Five KEGG pathways were enriched in high-CAS samples by performing GSEA. **(E)** Summary of the crosstalk of the subclusters. **(F)** Outgoing and incoming signaling of high- and low-CAS samples as well as other cell subclusters. **(G)** A heatmap illustrating the expression and the correlation between CAS and the pathways of interest.

We obtained the differentially expressed genes between high- and low-CAS samples and input the genes into Metascape, which is the online enrichment analysis tool. The significantly enriched terms in the high-CAS group are shown (**Figure 5A**). The top five items were matrix metalloproteinases, response to hexose, regulation of membrane potential, benzene-containing compound metabolic process, and steroid catabolic process. Moreover, the significantly enriched terms in the low-CAS group are shown (**Figure 5B**). The top five items were core matrisome, ECM organization, matrisome associated, proteoglycans, and cellular response to growth factor stimulus. In addition, we performed GSEA to explore the enriched pathways in high- and low-CAS samples (**Figures 5C, D**). We observed that ABC transporters, antigen processing and presentation, natural killer cell–mediated cytotoxicity, Nod-like receptor signaling pathway, and Toll-like receptor signaling pathway were enriched in the low-CAS group, whereas calcium signaling pathway, ECM receptor interaction, Notch signaling pathway, ribosome, and Vascular endothelial growth factor (VEGF) signaling pathway were enriched in the high-CAS samples. Then, according to the median expression of CAF-related genes, we divided the samples into two subgroups (high and low groups) and determined that the high groups had higher activated cell crosstalk than the low groups (**Figure 5E**). We summarized the significant crosstalk pathways, which indicated that the high group could activate most pathways (**Figure 5F**). Furthermore, we illustrated some specific pathways. On the one hand, the high group could send the signal from the Macrophage migration inhibitory factor (MIF) signaling pathway (CD74-CXCR4) (**Supplementary Figure 3A**), VEGF signaling pathway (VEGFA-VEGFR1) (**Supplementary Figure 3B**), PROS signaling pathway (PROS1-AXL) (**Supplementary Figure 3C**), and GDF signaling pathway (GDF15-TGFBR2) (**Supplementary Figure 3D**). On the other hand, the high group could receive signals from the Epidermal growth factor (EGF) signaling pathway (HBEGF-EGFR) (**Supplementary Figure 3E**) and Tumor necrosis factor-like weak inducer of apoptosis (TWEAK) signaling pathway (TNFSF12-TNFRSF12A) (**Supplementary Figure 3F**).

In addition, we analyzed some pathways of interest (**Figure 5G**). We illustrated that T cell coinhibition, angiogenesis, and Major histocompatibility complex class I were significantly upregulated in high-CAS samples. However, the type II Interferon (IFN) response pathway was significantly enriched in low-CAS samples. Moreover, the correlation between CAS and pathways is shown in the right panel. We found that T cell coinhibition had the most significantly positive correlation with CAS.

### Immune Analysis of the Constructed CAF-Related ANN Model

**Figure 6:**
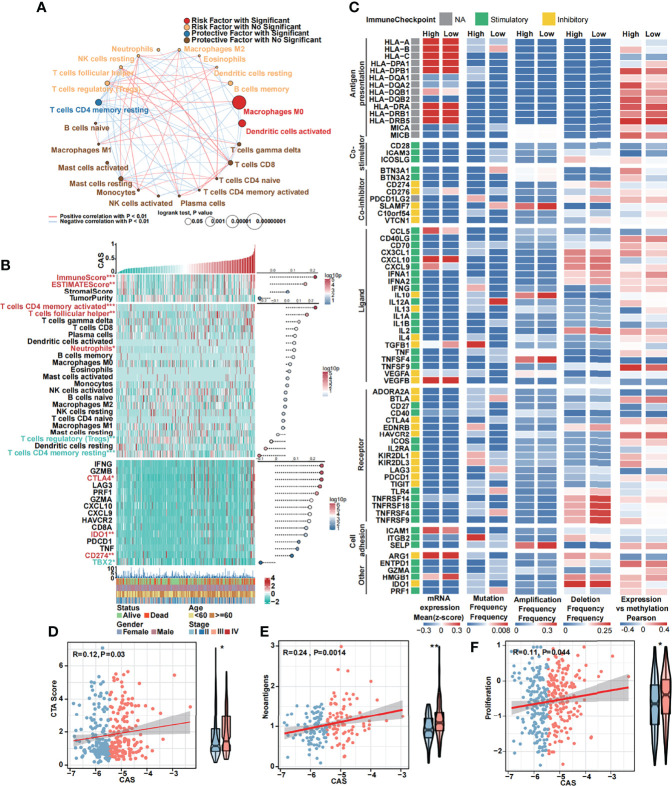
Immune analyses of the constructed ANN model. **(A)** Cox regression and Pearson’s correlation analysis of 22 TICs. **(B)** The expression and Pearson’s correlation of immune, ESTIMATE, stromal score, tumor purity, TICs, checkpoints, and immunocompetences of each sample are illustrated in a heatmap. **(C)** Multi-omics analysis of 75 immunomodulators between high- and low-CAS samples. **(D)** The Pearson’s correlation between the CTA score and CAS and the expression of the CTA score in high- and low-CAS samples. **(E)** The Pearson’s correlation between neoantigens and CAS, and the expression of neoantigens in high- and low-CAS samples. **(F)** The Pearson’s correlation between proliferation and CAS, and the expression of proliferation in high- and low-CAS samples.

The 22 TICs were divided into four groups based on the risk or protective factors with or without significance, and we also performed correlation analysis (**Figure 6A**). We demonstrated that M0 macrophages and activated dendritic cells were risk factors, whereas resting memory CD4 T cells were protective factors. We detected some positive or negative correlations among 22 TICs. Then, we illustrated a heatmap to analyze the TME and tumor purity and the expression of TICs and immunocompetence (**Figure 6B**). The immune and ESTIMATE scores were significantly higher in high-CAS samples and had a positive correlation with CAS. Activated memory CD4 T cells, follicular helper T cells, and neutrophils were highly expressed in high-CAS samples, whereas regulatory T cells and resting memory CD4 T cells were highly expressed in low-CAS samples, and CD8 T cells were highly regulated in patients with low CAS. To evaluate immunocompetence, we included immune checkpoints (*CD274*, *CTLA4*, *HAVCR2*, *IDO1*, *LAG3*, and *PDCD1*) and immunocompetence (*CD8A*, *CXCL10*, *CXCL9*, *GZMA*, *GZMB*, *IFNG*, *PRF1*, *TBX2*, and *TNF*) (23, 24). We found that the checkpoints *CTLA4*, *IDO1*, and *CD274* were highly expressed in high-CAS samples, whereas *TBX2* was significantly highly expressed in low-CAS samples. Furthermore, we performed a multi-omics analysis of 75 immunomodulators between high- and low-CAS samples. We included 14 antigen presentation factors, three co-stimulators, eight co-inhibitors, 22 ligands, 19 receptors, three cell adhesion factors, and six other factors (**Figure 6C**). We detected mRNA expression, frequency of mutation, amplification, and deletion, as well as the gene expression correlated with the DNA methylation beta value between high- and low-CAS samples. Finally, we detected cancer-testis antigen (CTA) (**Figure 6D**), neoantigens (**Figure 6E**), and proliferation (**Figure 6F**) scores between high- and low-CAS samples. The CTA score can increase the speed of tumorigenesis, against apoptosis, and enhance proliferation. We uncovered that high-CAS samples had higher CTA scores, neoantigen expression, and proliferation capacity. Through correlation analysis, we demonstrated a significantly positive correlation between CAS and the CTA score (P = 0.03), neoantigens (P = 0.0014), and proliferation (P= 0.044).

### Mutational Analysis of the Constructed CAF-Related ANN Model

**Figure 7:**
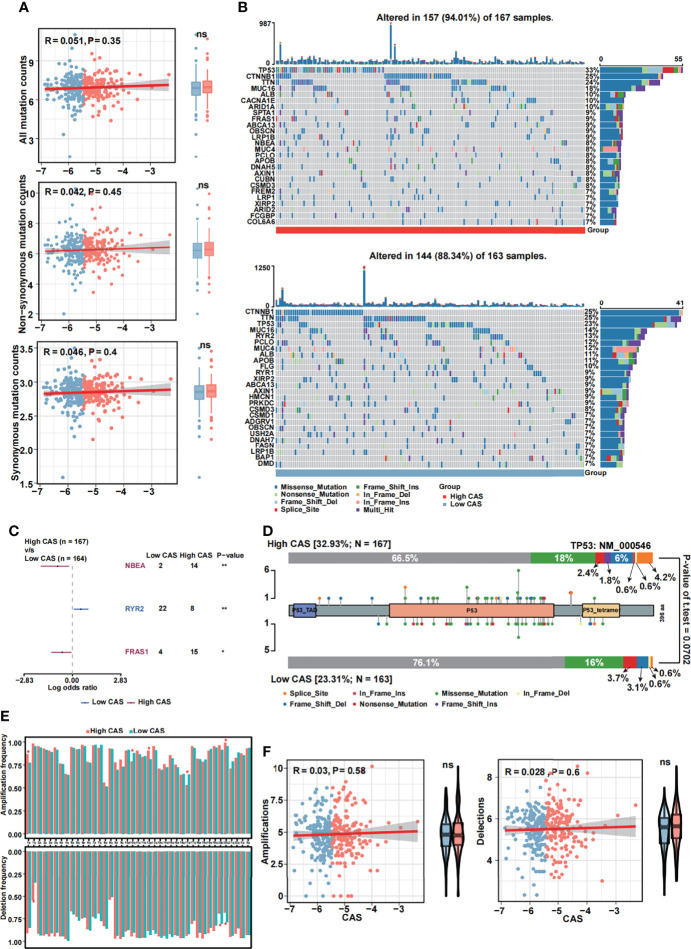
Mutational analyses of the constructed ANN model. **(A)** The Pearson’s correlation analysis between CAS and all mutation, non-synonymous mutation and synonymous mutation counts, and the counts of all mutation, non-synonymous mutation and synonymous mutation in high- and low-CAS samples. **(B)** Landscape of the 26 mutated genes that had more than 15 mutation counts in high-CAS samples (25) and low-CAS samples (lower). **(C)** Differentially mutated genes between high- and low-CAS samples. **(D)** A lollipop chart illustrates the location and the type of mutation in TP53. **(E)** The amplification and deletion frequency in each arm between high- and low-CAS samples. **(F)** The Pearson’s correlation between CAFs and amplification/deletion, and the frequency of amplification/deletion in high- and low-CAS samples. *P-value < 0.05; **P-value < 0.01; NS: No Significance.

We performed mutational analysis between low and high-CAS samples. First, we detected all mutation counts: non-synonymous and synonymous mutation counts (**Figure 7A**). Unfortunately, we did not detect any significance between the two groups. In addition, we filtered 26 genes whose mutation counts were more than 15 and subsequently illustrated a mutational landscape in high- and low-CAS samples (**Figure 7B**). The most frequently mutated gene in high-CAS samples was *TP53* (33%), followed by *CTNNB1* (25%) and *TTN* (24%). In comparison, the most frequently mutated gene in low-CAS samples was *CTNNB1* (25%), followed by *TTN* (25%) and *TP53* (23%). To determine differentially mutated genes between high- and low-CAS samples, we generated a forest plot (**Figure 7C**). The mutation counts of *NBEA* and *FRAS1* were higher in high-CAS samples, whereas *RYR2* had more mutation counts in low-CAS samples. Furthermore, we noticed that *TP53* had the highest percentage of mutation counts in high-CAS samples. Thus, we generated a lollipop chart of *TP53* to exhibit the mutation frequency and the types of mutation in high- and low-CAS samples (**Figure 7D**). After that, we generated a bar graph to illustrate the frequency of amplification and deletion of each arm in high- and low-CAS samples (**Figure 7E**). We demonstrated that the frequency of amplification in arms 1p, 12p, and 20q was significantly higher in high-CAS samples. However, in low-CAS samples, arms 10q and 10p showed a higher frequency of amplification. The frequency of deletion in arms 20q and 20p was significantly higher in low-CAS samples and lower in arm 1q compared to high-CAS samples. By performing correlation analysis between CAS and the frequency of amplification/deletion, unfortunately, we did not detect significance not only in the correlation but also in the frequency of mutation between high- and low-CAS samples.

### The Constructed CAF-Related ANN Model Guides Clinical Treatment

**Figure 8:**
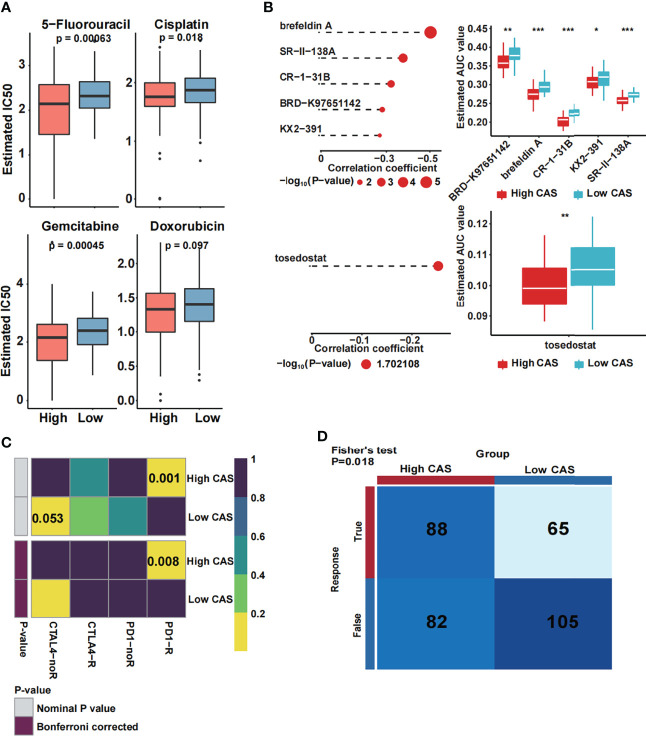
Prediction of drug and immune response. **(A)** The estimated IC50 of four common chemotherapeutic drugs in high- and low-CAS samples. **(B)** The prediction and the estimated AUC value of small-molecule drugs in the CTRP 2.0 and PRISM databases. **(C)** The immune response against PD1 and CTLA4 in patients with high and low CAS. **(D)** The total immune response in patients with high and low CAS. *P-value < 0.05; **P-value < 0.01; ***P-value < 0.001.

To our knowledge, one of the main treatments for HCC is chemotherapy, which includes 5-fluorouracil, cisplatin, gemcitabine, and doxorubicin. Thus, we predicted the sensitivity of the chemotherapeutic drugs between high- and low-CAS samples in the TCGA dataset (**Figure 8A**). We found that three drugs (5-fluorouracil, cisplatin, and gemcitabine) had more sensitivity in high-CAS samples than in low-CAS samples (P-value = 0.00063, 0.018, and 0.00045, respectively). We also detected the estimated IC50 between high- and low-CAS samples in the ICGC and GSE76427 datasets (**Supplementary Figures 4A, B**). 5-Fluorouracil, which was confirmed in three datasets, had higher sensitivity in high-CAS samples than in low-CAS samples. In addition, we predicted small-molecule drugs by using the CTRP and PRISM databases (**Figure 8B**). Brefeldin A, SR-II-138A, CR-1-31B, BRD-K97651142, KX2-391, and tosedostat were negatively correlated with the CAS, and the estimated AUC value was lower in high-CAS samples. The results indicated that the predicted small-molecule drugs had higher sensitivity in patients with high CAS. In addition, the immune response against PD1 and CTLA4 was predicted by using subclass mapping in the TCGA dataset (**Figure 8C**). We found that the immune response against PD1 was significant in patients with high CAS (P = 0.001). This result was confirmed in ICGC and GSE76427 datasets (P = 0.006 and 0.038) (**Supplementary Figures 4C, D**). Then, the total immune response was detected between high- and low-CAS samples by using the TIDE algorithm (**Figure 8D**). The results indicated that the patients with high CAS had a better immune response (P = 0.018). The result was also confirmed in the ICGC and GSE76427 datasets (**Supplementary Figures 4E, F**). Finally, other potential small molecular drugs and the corresponding mechanisms were illustrated by performing MoA analysis (**Supplementary Figure 5**).

## Discussion

In our study, we first identified 12 CAF-related genes by performing single-cell transcriptome, ligand–receptor interaction, Cox regression, and random forest analyses. A novel ANN model was then constructed, and the CAS of each sample was obtained. By performing functional analysis, we demonstrated that some cancer-related pathways and activated cell crosstalk pathways were enriched in high-CAS samples. In addition, we detected higher immunogenicity in high-CAS samples by performing an immune analysis. By illustrating the mutational landscape, we recovered significantly mutated genes between high- and low-CAS samples. Furthermore, a CAS-based nomogram was constructed for patients with HCC. The common chemotherapeutic drug 5-fluorouracil had been verified to have higher sensitivity in high-CAS samples in three cohorts. We also predicted some small-molecule drugs. Finally, we revealed that the immune response was better in high-CAS samples than in low-CAS samples.

We utilized the ANN model instead of the traditional Cox regression model. Here, we pointed out that the regression models have some limitations; for instance, the regression models are based on the presumption that all the data are linear, which is not always true in most biological situations (26). One of the advantages of the ANN model was that it can learn with experience and adapt to the error rate even in the situation that the validation cohorts were different from the derivation cohort, whereas the regression models can only fix within the parameters of the original derivation cohort. Another advantage is that the ANN model can handle large amounts of data (7). Thus, the ANN model was suitable for predicting the prognosis of patients.

We noticed that some cancer-related pathways, including the NOTCH signaling pathway and VEGF signaling pathway, were enriched in high-CAS samples. Interestingly, VEGF signaling was also activated in the high expression group in the cell communication analysis. Furthermore, according to the heatmap of the pathways of interest, we noticed that angiogenesis was enriched in high-CAS samples and positively correlated with CAS. The results demonstrated that the constructed ANN model was positively correlated with cancer progression and aggressive angiogenesis progression. One previous study revealed that CAFs promoted angiogenesis in HCC *via* the VEGF-mediated EZH2/VASH1 pathway (27). Another study indicated that CAFs promoted cancer invasion and the key function of CAFs was to drive vasculogenesis and angiogenesis (28). Thus, anti-VEGF therapy might become a potential method for patients with HCC with high CAS. In addition, we recovered that the type II IFN (IFN-γ) response pathway was enriched in low-CAS samples. A previous study revealed that IFN-γ was a vital factor in tumor cell elimination (29), and this finding was supported by experiments using a mouse model (30).

According to the heatmap, the immune score and ESTIMATE score were significantly higher in high-CAS samples, revealing that our ANN model was associated with immune regulation. In addition, memory CD4^+^ T cells undergo fast expansion and cause a more effective and faster immune response (31). Follicular helper T cells are a subset of CD4^+^ cells that play a critical role in the immune and effector response functions of T cells (32). In our study, we demonstrated that activated memory CD4^+^ T cells and follicular helper T cells were highly expressed in high-CAS samples and had a positive correlation with CAS, which indicated that high-CAS samples had a better immune response than low-CAS samples. Studies have found that neutrophils are involved in the different stages of the oncogenic process, including tumor initiation, growth, proliferation, and metastatic spreading (33, 34). The expression of neutrophils was significantly higher in high-CAS samples, and the result was consistent with previous studies. Moreover, the immune checkpoints *CTLA4*, *IDO1*, and *CD274* were increased in the high-CAS samples. Previous studies have pointed out that checkpoints are key players in cancer development and immunotherapy (35). Thus, blocking the expression of *CTLA4*, *IDO1*, and *CD274* may be a novel target for the immune treatment of HCC. The CTA, neoantigen, and proliferation scores were positively correlated with CAS, which was consistent with our results.

By performing mutational analysis, we revealed that patients with high CAS had a higher mutational frequency of *NBEA* and *FRAS1* and a lower mutational frequency of *RYR2*. *NBEA* has been certified as a novel tumor suppressor gene, and mutation of *NBEA* can cause poor outcomes in multiple myeloma (36). Previous research found that *RYR2* mutation was significantly associated with better clinical prognosis (37) and reduced the risk of development (38) in breast cancer. In addition, the *RYR2* mutation correlated with better prognosis was involved in the immune response and enhanced antitumor immunity in esophageal adenocarcinoma (18). *FRAS1* was found to have a high mutational frequency in high-CAS samples. A previous study indicated that *FRAS1* has the ability to regulate epidermal basement membrane adhesion and cell migration (39). In addition, *FRAS1* was more frequently mutated in metastatic breast cancer than in primary breast cancer (40). Another study demonstrated that *FRAS1* mutation may be associated with an increase in the development of metastatic disease or death from prostate cancer (41). Above all, the results were coincidental with our findings. Moreover, we demonstrated that the frequency of *TP53* mutation is the highest in patients with high CAS (33%). *TP53* acts as a tumor suppressor and induces growth inhibition and apoptosis (42). Approximately, 13%–48% of liver cancers harbor *TP53* mutations (43, 44). Our findings and the results from previous studies were consistent.

We predicted some small-molecule drugs with higher sensitivity in patients with high CAS, including brefeldin A, SR-II-138A, CR-1-31B, BRD-K97651142, KX2-391, and tosedostat. Brefeldin A has been reported to markedly inhibit proliferation and induce autophagic cell death *via* the Akt/mTOR and ERK pathways when encapsulated in mixed nanomicelles (45). CR-1-31B, an inhibitor of eukaryotic translation initiation factor 4A, has been found to significantly reduce the growth and initiate the apoptosis of gallbladder cancer cells (46). In addition, one article reported that the Src/FAK pathway inhibitor KX2-391 significantly increased the sensitivity of HepG2/doxorubicin cells to doxorubicin in HCC (47). The novel metalloenzyme inhibitor tosedostat has shown promising activity for patients with acute myeloid leukemia (48). However, we have not found any reports about SR-II-138A and BRD-K97651142.

In our study, we first constructed an ANN model based on CAF-related prognostic genes in HCC. However, this study still has some limitations. To begin with, our data were obtained from the TCGA, ICGC, and GEO online databases, which need to be verified in a large sample in reality. In addition, the prognostic ANN model needs to be certified in a real clinical cohort before application.

In conclusion, we created a novel CAF-related ANN model that is suitable for individually predicting the prognosis of patients with HCC and guiding clinical treatment through functional, mutational, immune, and clinical analyses.

## Data Availability Statement

The original contributions presented in the study are included in the article/**Supplementary Material**. Further inquiries can be directed to the corresponding author.

## Author Contributions

YL and HT: Conception and design and writing of the manuscript. TY and JT: Development of methodology, and analysis and interpretation of the data. HS: Review and revision of the manuscript. All authors contributed to the article and approved the submitted version.

## Conflict of Interest

The authors declare that the research was conducted in the absence of any commercial or financial relationships that could be construed as a potential conflict of interest.

## Publisher’s Note

All claims expressed in this article are solely those of the authors and do not necessarily represent those of their affiliated organizations, or those of the publisher, the editors and the reviewers. Any product that may be evaluated in this article, or claim that may be made by its manufacturer, is not guaranteed or endorsed by the publisher.

## Supplementary Material

Supplementary Figure 1 Validation of the constructed ANN model in ICGC and GSE76427. **(A)** The CAS and corresponding survival status in each sample in the ICGC dataset. **(B)** The CAS and corresponding survival status in each sample in the GSE76427 dataset. **(C)** Kaplan-Meier analysis of high and low CAS samples in the ICGC dataset. **(D)** Kaplan-Meier analysis of high and low CAS samples in the GSE76427 dataset. **(E)** ROC analysis of the ANN model in 1-, 3-, and 5-year survival prediction in the ICGC dataset. **(F)** ROC analysis of the ANN model in 1-, 3-, and 5-year survival prediction in the GSE76427 dataset.

Supplementary Figure 2 Univariate Cox regression subgroup analyses of the CAS in three datasets. **(A)** Subgroup analysis in TCGA dataset. **(B)** Subgroup analysis in the ICGC dataset. **(C)** Subgroup analysis in the GSE76427 dataset.

Supplementary Figure 3 The different ligand–receptor pairs between high and low CAS samples. **(A)** High CAS cells communicate with TAMs, B cells and T cells through CD74-CXCR4 in the MIF signaling pathway. **(B)** High CAS cells communicate with TECs *via* VEGFA-VEGFR1 in the VEGF signaling pathway. **(C)** High CAS cells communicate with TAMs through TNFSF12-TNFRSF12A in the TWEAK signaling pathway. **(D)** High CAS cells communicate with TAMs and CAFs through PROS1-AXL in the PROS signaling pathway. **(E)** High CAS cells communicate with TAMs through HBEGF-EGFR in the EGF signaling pathway. **(F)** High CAS cells communicate with TECs and TAMs through GDF15-TGFBR2 in the GDF signaling pathway.

Supplementary Figure 4 Validation of the sensitivity of the chemotherapeutic drugs and immune response. **(A)** The estimated IC50 of four common chemotherapeutic drugs in high and low CAS samples in the ICGC dataset. **(B)** The estimated IC50 of four common chemotherapeutic drugs in high and low CAS samples in GSE76427. **(C)** The immune response against PD1 and CTLA4 in high and low CAS patients in the ICGC dataset. **(D)** The immune response against PD1 and CTLA4 in high and low CAS patients in the GSE76427 dataset. **(E)** The total immune response in high and low CAS patients in the ICGC dataset. **(F)** The total immune response in high and low CAS patients in the GSE76427 dataset.

Supplementary Figure 5 Prediction of the possible drugs and the corresponding mechanism by MoA analysis.

## References

[1] El-Serag HB Rudolph KL. Hepatocellular Carcinoma: Epidemiology and Molecular Carcinogenesis. Gastroenterology (2007) 132(7):2557–76. doi: 10.1053/j.gastro.2007.04.061 17570226

[2] Fitzmaurice C Allen C Barber RM Barregard L Bhutta ZA Brenner H. Global, Regional, and National Cancer Incidence, Mortality, Years of Life Lost, Years Lived With Disability, and Disability-Adjusted Life-Years for 32 Cancer Groups, 1990 to 2015: A Systematic Analysis for the Global Burden of Disease Study. JAMA Oncol (2017) 3(4):524–48. doi: 10.1001/jamaoncol.2016.5688 PMC6103527 27918777

[3] Thomas MB Jaffe D Choti MM Belghiti J Curley S Fong Y. Hepatocellular Carcinoma: Consensus Recommendations of the National Cancer Institute Clinical Trials Planning Meeting. J Clin Oncol Off J Am Soc Clin Oncol (2010) 28(25):3994–4005. doi: 10.1200/JCO.2010.28.7805 PMC2940397 20679622

[4] Nurmik M Ullmann P Rodriguez F Haan S Letellier E. In Search of Definitions: Cancer-Associated Fibroblasts and Their Markers. Int J Cancer (2020) 146(4):895–905. doi: 10.1002/ijc.32193 30734283 PMC6972582

[5] Kalluri R. The Biology and Function of Fibroblasts in Cancer. Nat Rev Cancer (2016) 16(9):582–98. doi: 10.1038/nrc.2016.73 27550820

[6] Lee JG Jun S Cho YW Lee H Kim GB Seo JB. Deep Learning in Medical Imaging: General Overview. Korean J Radiol (2017) 18(4):570–84. doi: 10.3348/kjr.2017.18.4.570 PMC5447633 28670152

[7] Ghoshal UC Das A. Models for Prediction of Mortality From Cirrhosis With Special Reference to Artificial Neural Network: A Critical Review. Hepatol Int (2008) 2(1):31–8. doi: 10.1007/s12072-007-9026-1 PMC2716874 19669277

[8] Yazdani Charati J Janbabaei G Alipour N Mohammadi S Ghorbani Gholiabad S Fendereski A. Survival Prediction of Gastric Cancer Patients by Artificial Neural Network Model. Gastroenterol Hepatol Bed Bench (2018) 11(2):110–7. PMC5990918 29910851

[9] Afshar S Afshar S Warden E Manochehri H Saidijam M. Application of Artificial Neural Network in miRNA Biomarker Selection and Precise Diagnosis of Colorectal Cancer. Iran BioMed J (2019) 23(3):175–83. doi: 10.29252/ibj.23.3.175 PMC6462295 30056689

[10] Ma L Wang L Khatib SA Chang CW Heinrich S Dominguez DA. Single-Cell Atlas of Tumor Cell Evolution in Response to Therapy in Hepatocellular Carcinoma and Intrahepatic Cholangiocarcinoma. J Hepatol (2021) 75(6):1397–408. doi: 10.1016/j.jhep.2021.06.028 PMC8604764 34216724

[11] Qiu X Mao Q Tang Y Wang L Chawla R Pliner HA. Reversed Graph Embedding Resolves Complex Single-Cell Trajectories. Nat Methods (2017) 14(10):979–82. doi: 10.1038/nmeth.4402 PMC5764547 28825705

[12] Liang JY Wang DS Lin HC Chen XX Yang H Zheng Y. A Novel Ferroptosis-Related Gene Signature for Overall Survival Prediction in Patients With Hepatocellular Carcinoma. Int J Biol Sci (2020) 16(13):2430–41. doi: 10.7150/ijbs.45050 PMC7378635 32760210

[13] McDermott DF Huseni MA Atkins MB Motzer RJ Rini BI Escudier B. Clinical Activity and Molecular Correlates of Response to Atezolizumab Alone or in Combination With Bevacizumab Versus Sunitinib in Renal Cell Carcinoma. Nat Med (2018) 24(6):749–57. doi: 10.1038/s41591-018-0053-3 PMC6721896 29867230

[14] Gibbons DL Creighton CJ. Pan-Cancer Survey of Epithelial-Mesenchymal Transition Markers Across the Cancer Genome Atlas. Dev Dyn (2018) 247(3):555–64. doi: 10.1002/dvdy.24485 PMC5503821 28073171

[15] Liberzon A Subramanian A Pinchback R Thorvaldsdóttir H Tamayo P Mesirov JP. Molecular Signatures Database (MSigDB) 3. 0 Bioinf (Oxford England) (2011) 27(12):1739–40. doi: 10.1093/bioinformatics/btr260 PMC3106198 21546393

[16] Thorsson V Gibbs DL Brown SD Wolf D Bortone DS Ou Yang TH. The Immune Landscape of Cancer. Immunity (2018) 48(4):812–30.e14. doi: 10.1016/j.immuni.2018.03.023 29628290 PMC5982584

[17] Jiang P Gu S Pan D Fu J Sahu A Hu X. Signatures of T Cell Dysfunction and Exclusion Predict Cancer Immunotherapy Response. Nat Med (2018) 24(10):1550–8. doi: 10.1038/s41591-018-0136-1 PMC6487502 30127393

[18] Liu Z Liu L Jiao D Guo C Wang L Li Z. Association of RYR2 Mutation With Tumor Mutation Burden, Prognosis, and Antitumor Immunity in Patients With Esophageal Adenocarcinoma. Front Genet (2021) 12. doi: 10.3389/fgene.2021.669694 PMC8166246 34079583

[19] Liu Z Lu T Wang L Liu L Li L Han X. Comprehensive Molecular Analyses of a Novel Mutational Signature Classification System With Regard to Prognosis, Genomic Alterations, and Immune Landscape in Glioma. Front Mol Biosci (2021) 8:682084. doi: 10.3389/fmolb.2021.682084 34307451 PMC8293748

[20] Liu Z Lu T Li J Wang L Xu K Dang Q. Clinical Significance and Inflammatory Landscape of Anovel Recurrence-Associated Immune Signature in Stage II/III Colorectal Cancer. Front Immunol (2021) 12. doi: 10.3389/fimmu.2021.702594 PMC8358813 34394098

[21] Roh W Chen PL Reuben A Spencer CN Prieto PA Miller JP. Integrated Molecular Analysis of Tumor Biopsies on Sequential CTLA-4 and PD-1 Blockade Reveals Markers of Response and Resistance. Sci Transl Med (2017) 9(379). doi: 10.1126/scitranslmed.aah3560 PMC5819607 28251903

[22] Meistermann D Bruneau A Loubersac S Reignier A Firmin J François-Campion V. Integrated Pseudotime Analysis of Human Pre-Implantation Embryo Single-Cell Transcriptomes Reveals the Dynamics of Lineage Specification. Cell Stem Cell (2021) 28(9):1625–40.e6. doi: 10.1016/j.stem.2021.04.027 34004179

[23] Ayers M Lunceford J Nebozhyn M Murphy E Loboda A Kaufman DR. IFN-γ-Related mRNA Profile Predicts Clinical Response to PD-1 Blockade. J Clin Invest (2017) 127(8):2930–40. doi: 10.1172/JCI91190 PMC5531419 28650338

[24] Hugo W Zaretsky JM Sun L Song C Moreno BH Hu-Lieskovan S. Genomic and Transcriptomic Features of Response to Anti-PD-1 Therapy in Metastatic Melanoma. Cell (2016) 165(1):35–44. doi: 10.1016/j.cell.2016.02.065 26997480 PMC4808437

[25] Wei T Weiler SME Tóth M Sticht C Lutz T Thomann S. YAP-Dependent Induction of UHMK1 Supports Nuclear Enrichment of the Oncogene MYBL2 and Proliferation in Liver Cancer Cells. Oncogene (2019) 38(27):5541–50. doi: 10.1038/s41388-019-0801-y 30936457

[26] Cross SS Harrison RF Kennedy RL. Introduction to Neural Networks. Lancet (London England) (1995) 346(8982):1075–9. doi: 10.1016/S0140-6736(95)91746-2 7564791

[27] Huang B Huang M Li Q. Cancer-Associated Fibroblasts Promote Angiogenesis of Hepatocellular Carcinoma by VEGF-Mediated EZH2/VASH1 Pathway. Technol Cancer Res Treat (2019) 18:1533033819879905. doi: 10.1177/1533033819879905 31757187 PMC6876164

[28] Pape J Magdeldin T Stamati K Nyga A Loizidou M Emberton M. Cancer-Associated Fibroblasts Mediate Cancer Progression and Remodel the Tumouroid Stroma. Br J Cancer (2020) 123(7):1178–90. doi: 10.1038/s41416-020-0973-9 PMC7524802 32641866

[29] Dighe AS Richards E Old LJ Schreiber RD. Enhanced *In Vivo* Growth and Resistance to Rejection of Tumor Cells Expressing Dominant Negative IFN Gamma Receptors. Immunity (1994) 1(6):447–56. doi: 10.1016/1074-7613(94)90087-6 7895156

[30] Kaplan DH Shankaran V Dighe AS Stockert E Aguet M Old LJ. Demonstration of an Interferon Gamma-Dependent Tumor Surveillance System in Immunocompetent Mice. Proc Natl Acad Sci USA (1998) 95(13):7556–61. doi: 10.1073/pnas.95.13.7556 PMC22681 9636188

[31] Golubovskaya V Wu L. Different Subsets of T Cells, Memory, Effector Functions, and CAR-T Immunotherapy. Cancers (2016) 8(3). doi: 10.3390/cancers8030036 PMC4810120 26999211

[32] Raphael I Nalawade S Eagar TN Forsthuber TG. T Cell Subsets and Their Signature Cytokines in Autoimmune and Inflammatory Diseases. Cytokine (2015) 74(1):5–17. doi: 10.1016/j.cyto.2014.09.011 25458968 PMC4416069

[33] Swierczak A Mouchemore KA Hamilton JA Anderson RL. Neutrophils: Important Contributors to Tumor Progression and Metastasis. Cancer Metastasis Rev (2015) 34(4):735–51. doi: 10.1007/s10555-015-9594-9 26361774

[34] Coffelt SB Wellenstein MD de Visser KE. Neutrophils in Cancer: Neutral No More. Nat Rev Cancer (2016) 16(7):431–46. doi: 10.1038/nrc.2016.52 27282249

[35] Lian J Yue Y Yu W Zhang Y. Immunosenescence: A Key Player in Cancer Development. J Hematol Oncol (2020) 13(1):151. doi: 10.1186/s13045-020-00986-z 33168037 PMC7653700

[36] O'Neal J Gao F Hassan A Monahan R Barrios S Kilimann MW. Neurobeachin (NBEA) Is a Target of Recurrent Interstitial Deletions at 13q13 in Patients With MGUS and Multiple Myeloma. Exp Hematol (2009) 37(2):234–44. doi: 10.1016/j.exphem.2008.10.014 PMC2868587 19135901

[37] Xu Z Xiang L Wang R Xiong Y Zhou H Gu H. Bioinformatic Analysis of Immune Significance of RYR2 Mutation in Breast Cancer. BioMed Res Int (2021) 2021:8072796. doi: 10.1155/2021/8072796 34888385 PMC8651385

[38] Wei Y Wang X Zhang Z Zhao C Chang Y Bian Z. Impact of NR5A2 and RYR2 3'UTR Polymorphisms on the Risk of Breast Cancer in a Chinese Han Population. Breast Cancer Res Treat (2020) 183(1):1–8. doi: 10.1007/s10549-020-05736-w 32572717

[39] Kiyozumi D Sugimoto N Sekiguchi K. Breakdown of the Reciprocal Stabilization of QBRICK/Frem1, Fras1, and Frem2 at the Basement Membrane Provokes Fraser Syndrome-Like Defects. Proc Natl Acad Sci USA (2006) 103(32):11981–6. doi: 10.1073/pnas.0601011103 PMC1567684 16880404

[40] Lefebvre C Bachelot T Filleron T Pedrero M Campone M Soria JC. Mutational Profile of Metastatic Breast Cancers: A Retrospective Analysis. PLoS Med (2016) 13(12):e1002201. doi: 10.1371/journal.pmed.1002201 28027327 PMC5189935

[41] Wang V Geybels MS Jordahl KM Gerke T Hamid A Penney KL. A Polymorphism in the Promoter of FRAS1 Is a Candidate SNP Associated With Metastatic Prostate Cancer. Prostate (2021) 81(10):683–93. doi: 10.1002/pros.24148 PMC8491321 33956343

[42] Belinky F Nativ N Stelzer G Zimmerman S Iny Stein T Safran M. PathCards: Multi-Source Consolidation Hum Biol Pathways Database (Oxford) (2015) 2015. doi: 10.1093/database/bav006 PMC4343183 25725062

[43] Ahn SM Jang SJ Shim JH Kim D Hong SM Sung CO. Genomic Portrait of Resectable Hepatocellular Carcinomas: Implications of RB1 and FGF19 Aberrations for Patient Stratification. Hepatology (2014) 60(6):1972–82. doi: 10.1002/hep.27198 24798001

[44] Takai A Dang HT Wang XW. Identification of Drivers From Cancer Genome Diversity in Hepatocellular Carcinoma. Int J Mol Sci (2014) 15(6):11142–60. doi: 10.3390/ijms150611142 PMC4100204 24955791

[45] Zhang JM Jiang YY Huang QF Lu XX Wang GH Shao CL. Brefeldin A Delivery Nanomicelles in Hepatocellular Carcinoma Therapy: Characterization, Cytotoxic Evaluation *In Vitro*, and Antitumor Efficiency *In Vivo* . Pharmacol Res (2021) 172:105800. doi: 10.1016/j.phrs.2021.105800 34363949

[46] Cao Y He Y Yang L Luan Z. Targeting Eif4a Using Rocaglate CR−1−31B Sensitizes Gallbladder Cancer Cells to TRAIL−Mediated Apoptosis Through the Translational Downregulation of C−FLIP. Oncol Rep (2021) 45(1):230–8. doi: 10.1016/j.biopha.2017.09.065 PMC7709814 33416145

[47] Yu M Zou Q Wu X Han G Tong X. Connexin 32 Affects Doxorubicin Resistance in Hepatocellular Carcinoma Cells Mediated by Src/FAK Signaling Pathway. Biomedicine Pharmacotherapy = Biomedecine pharmacotherapie (2017) 95:1844–52. doi: 10.1016/j.biopha.2017.09.065 28968929

[48] DiNardo CD Cortes JE. Tosedostat for the Treatment of Relapsed and Refractory Acute Myeloid Leukemia. Expert Opin Investig Drugs (2014) 23(2):265–72. doi: 10.1517/13543784.2014.864276 24313331

