# Supplementary figures and images for: A Novel Artificial Neural Network Prognostic Model Based on a Cancer-Associated Fibroblast Activation Score System in Hepatocellular Carcinoma
Source: Front Immunol. 2022 Jul 8;13:927041. doi: 10.3389/fimmu.2022.927041 (PMC9304772; doi:10.3389/fimmu.2022.927041)

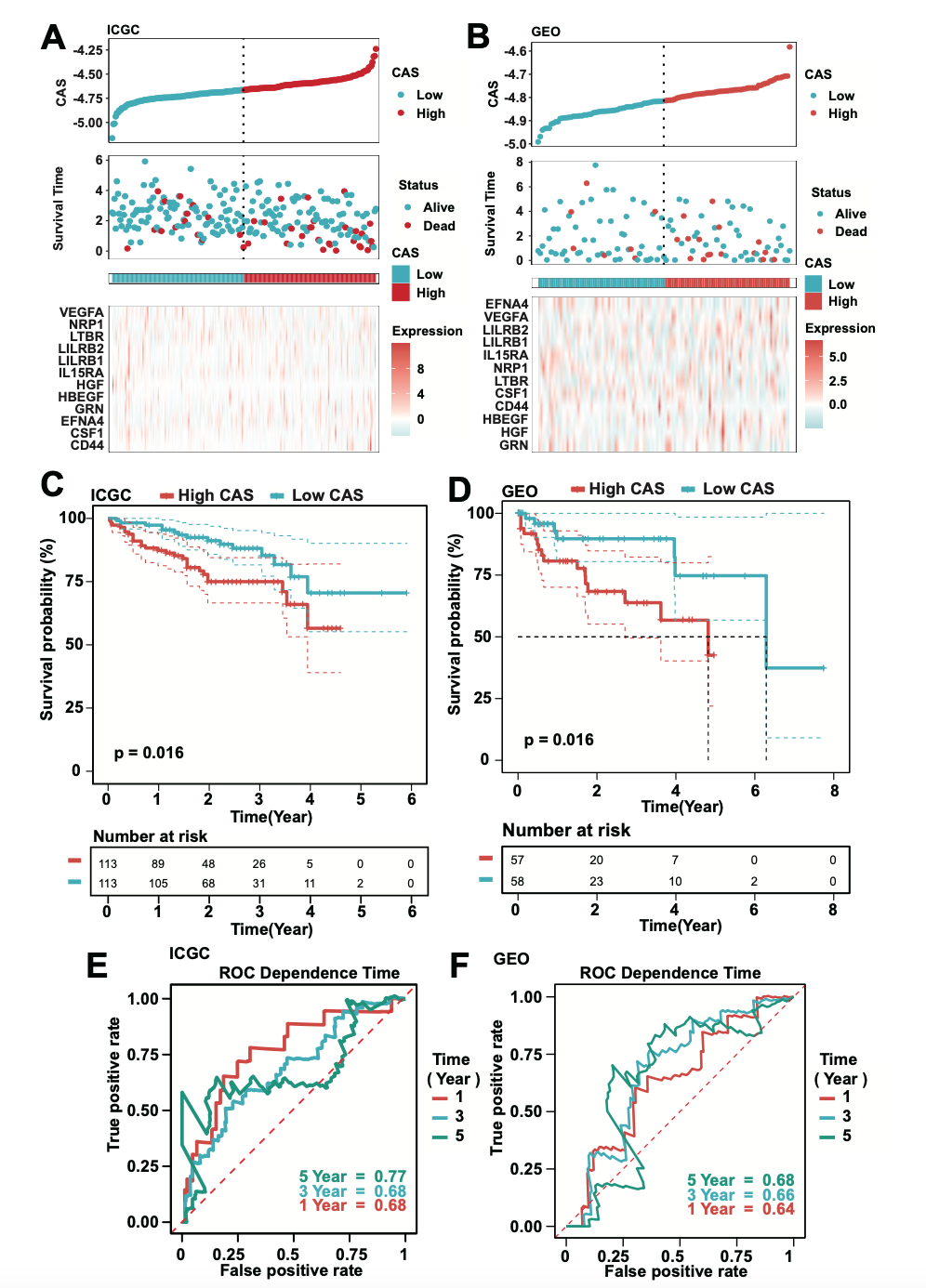

Supplement: Supplementary Figure 1 — Validation of the constructed ANN model in ICGC and GSE76427. (A) The CAS and corresponding survival status in each sample in the ICGC dataset. (B) The CAS and corresponding survival status in each sample in the GSE76427 dataset. (C) Kaplan-Meier analysis of high and low CAS samples in the ICGC dataset. (D) Kaplan-Meier analysis of high and low CAS samples in the GSE76427 dataset. (E) ROC analysis of the ANN model in 1-, 3-, and 5-year survival prediction in the ICGC dataset. (F) ROC analysis of the ANN model in 1-, 3-, and 5-year survival prediction in the GSE76427 dataset. [file Image_1.tif]

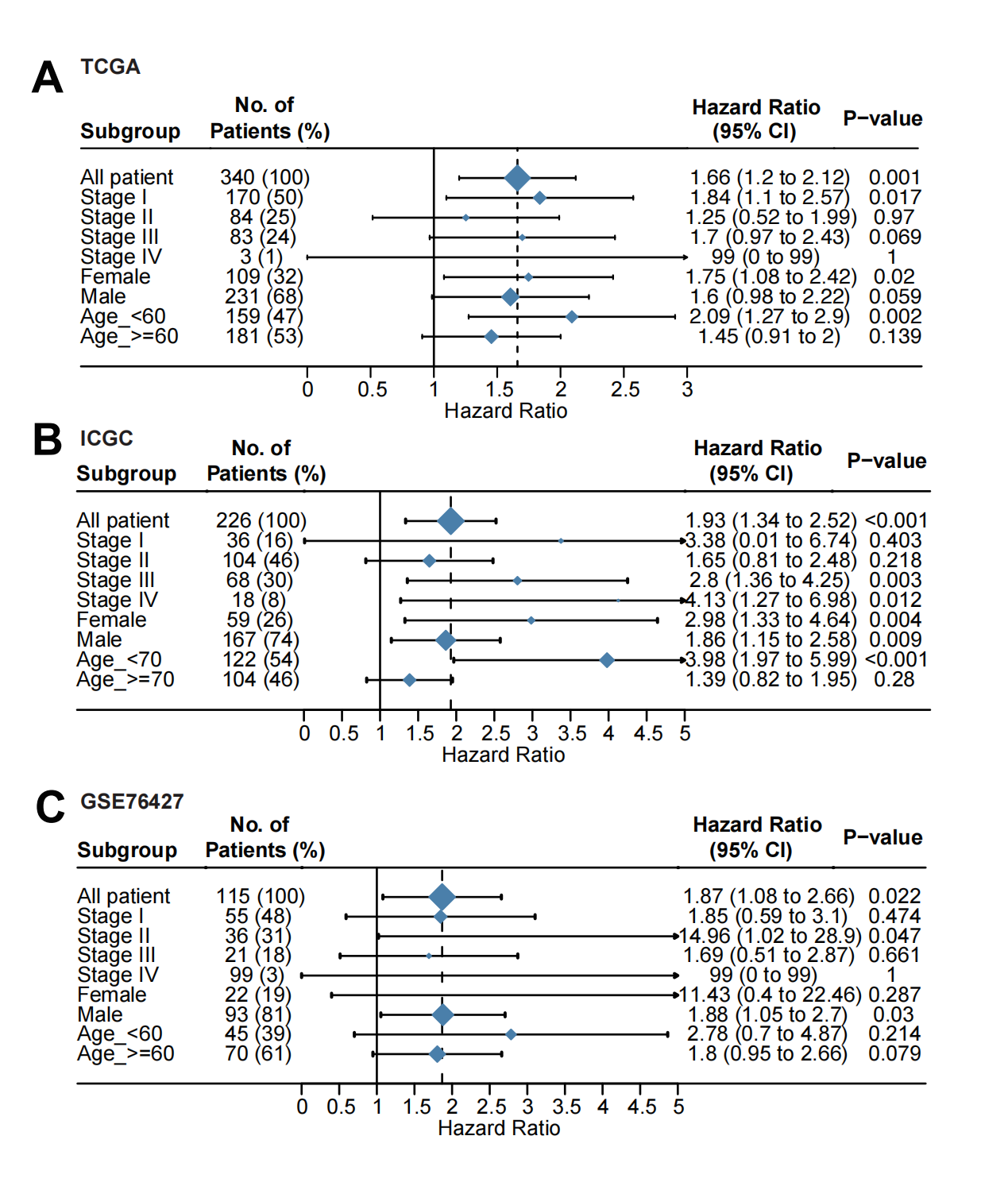

Supplement: Supplementary Figure 2 — Univariate Cox regression subgroup analyses of the CAS in three datasets. (A) Subgroup analysis in TCGA dataset. (B) Subgroup analysis in the ICGC dataset. (C) Subgroup analysis in the GSE76427 dataset. [file Image_2.tif]

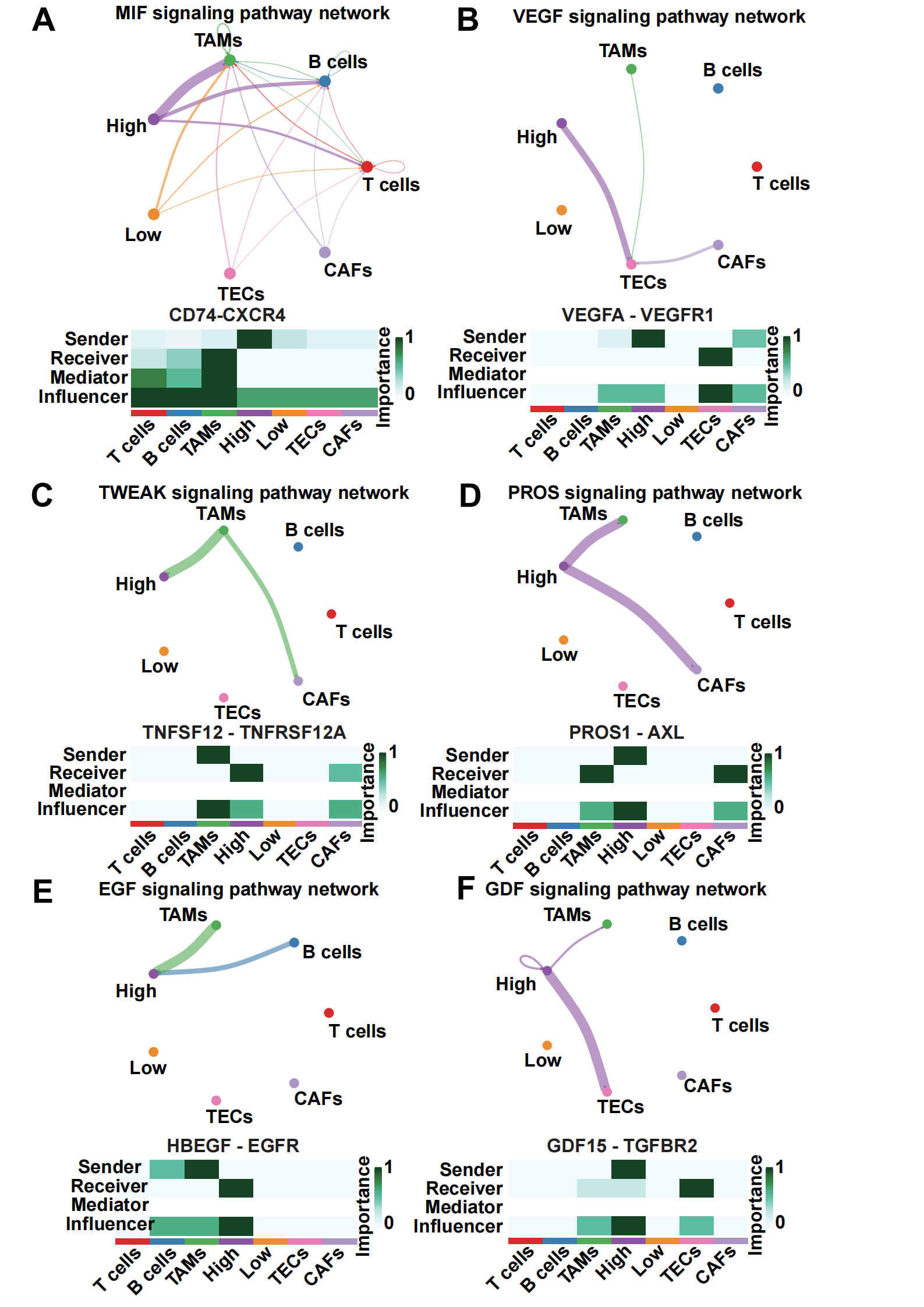

Supplement: Supplementary Figure 3 — The different ligand–receptor pairs between high and low CAS samples. (A) High CAS cells communicate with TAMs, B cells and T cells through CD74-CXCR4 in the MIF signaling pathway. (B) High CAS cells communicate with TECs via VEGFA-VEGFR1 in the VEGF signaling pathway. (C) High CAS cells communicate with TAMs through TNFSF12-TNFRSF12A in the TWEAK signaling pathway. (D) High CAS cells communicate with TAMs and CAFs through PROS1-AXL in the PROS signaling pathway. (E) High CAS cells communicate with TAMs through HBEGF-EGFR in the EGF signaling pathway. (F) High CAS cells communicate with TECs and TAMs through GDF15-TGFBR2 in the GDF signaling pathway. [file Image_3.tif]

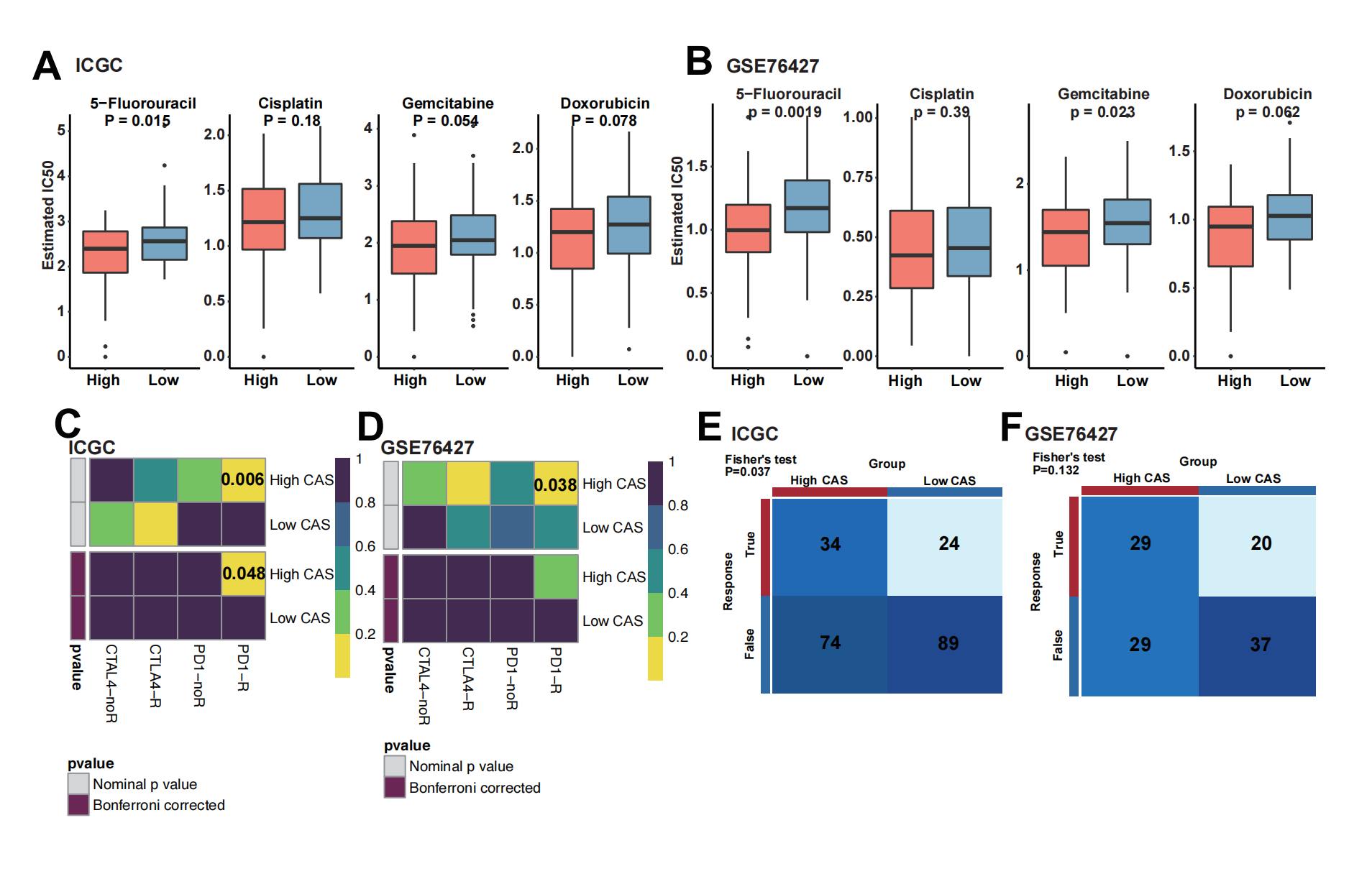

Supplement: Supplementary Figure 4 — Validation of the sensitivity of the chemotherapeutic drugs and immune response. (A) The estimated IC50 of four common chemotherapeutic drugs in high and low CAS samples in the ICGC dataset. (B) The estimated IC50 of four common chemotherapeutic drugs in high and low CAS samples in GSE76427. (C) The immune response against PD1 and CTLA4 in high and low CAS patients in the ICGC dataset. (D) The immune response against PD1 and CTLA4 in high and low CAS patients in the GSE76427 dataset. (E) The total immune response in high and low CAS patients in the ICGC dataset. (F) The total immune response in high and low CAS patients in the GSE76427 dataset. [file Image_4.tif]

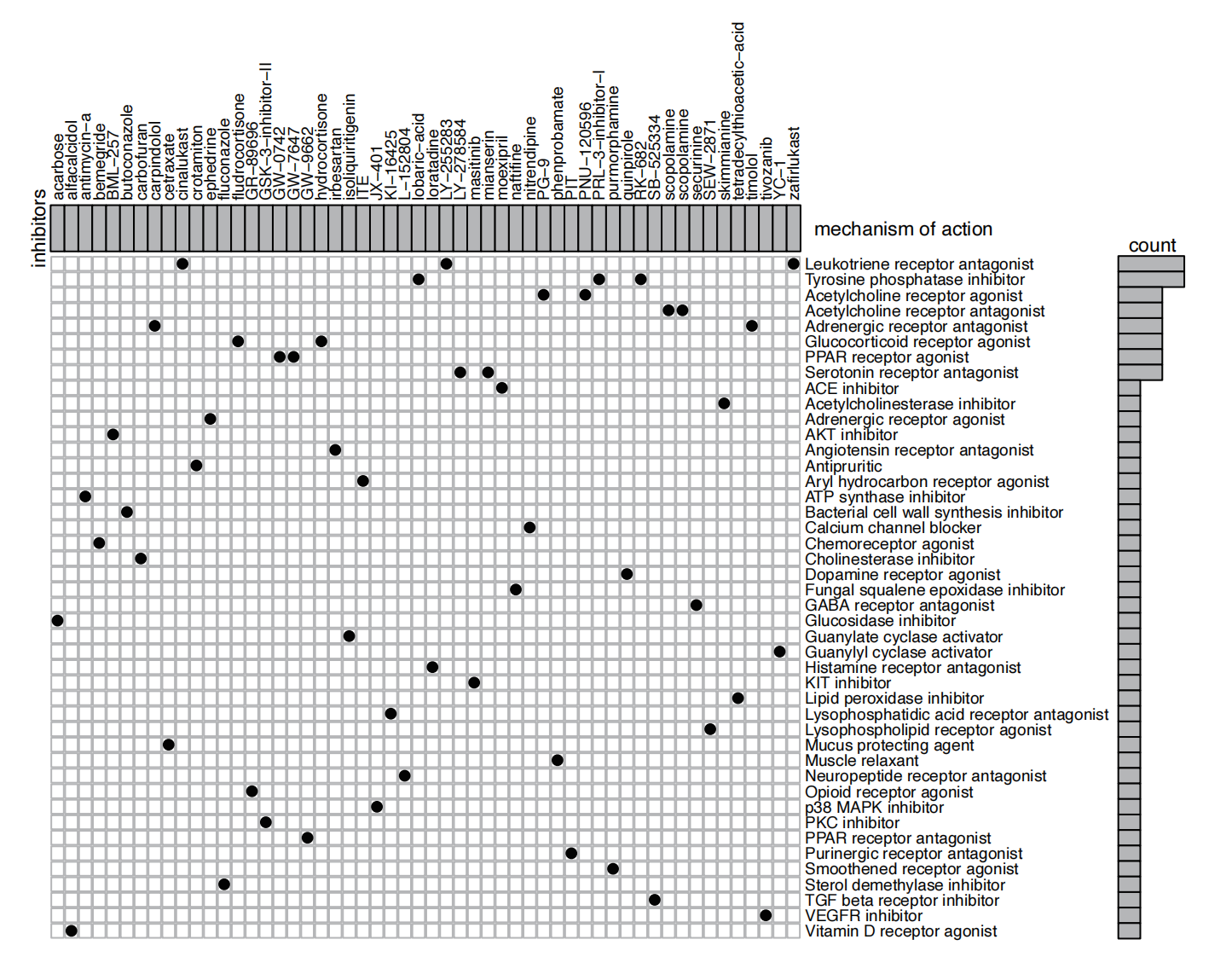

Supplement: Supplementary Figure 5 — Prediction of the possible drugs and the corresponding mechanism by MoA analysis. [file Image_5.tif]
